# Amiloride-sensitive channels in type I fungiform taste cells in mouse

**DOI:** 10.1186/1471-2202-9-1

**Published:** 2008-01-02

**Authors:** Aurelie Vandenbeuch, Tod R Clapp, Sue C Kinnamon

**Affiliations:** 1 Department of Biomedical Science, Colorado State University, Fort Collins, USA

## Abstract

**Background:**

Taste buds are the sensory organs of taste perception. Three types of taste cells have been described. Type I cells have voltage-gated outward currents, but lack voltage-gated inward currents. These cells have been presumed to play only a support role in the taste bud. Type II cells have voltage-gated Na^+ ^and K^+ ^current, and the receptors and transduction machinery for bitter, sweet, and umami taste stimuli. Type III cells have voltage-gated Na^+^, K^+^, and Ca^2+ ^currents, and make prominent synapses with afferent nerve fibers. Na^+ ^salt transduction in part involves amiloride-sensitive epithelial sodium channels (ENaCs). In rodents, these channels are located in taste cells of fungiform papillae on the anterior part of the tongue innervated by the chorda tympani nerve. However, the taste cell type that expresses ENaCs is not known. This study used whole cell recordings of single fungiform taste cells of transgenic mice expressing GFP in Type II taste cells to identify the taste cells responding to amiloride. We also used immunocytochemistry to further define and compare cell types in fungiform and circumvallate taste buds of these mice.

**Results:**

Taste cell types were identified by their response to depolarizing voltage steps and their presence or absence of GFP fluorescence. TRPM5-GFP taste cells expressed large voltage-gated Na^+ ^and K^+ ^currents, but lacked voltage-gated Ca^2+ ^currents, as expected from previous studies. Approximately half of the unlabeled cells had similar membrane properties, suggesting they comprise a separate population of Type II cells. The other half expressed voltage-gated outward currents only, typical of Type I cells. A single taste cell had voltage-gated Ca^2+ ^current characteristic of Type III cells. Responses to amiloride occurred only in cells that lacked voltage-gated inward currents. Immunocytochemistry showed that fungiform taste buds have significantly fewer Type II cells expressing PLC signalling components, and significantly fewer Type III cells than circumvallate taste buds.

**Conclusion:**

The principal finding is that amiloride-sensitive Na^+ ^channels appear to be expressed in cells that lack voltage-gated inward currents, likely the Type I taste cells. These cells were previously assumed to provide only a support function in the taste bud.

## Background

At the peripheral taste system level, it is still unclear whether each taste quality is transduced by a separate population of taste cells, each connected to distinct nerve fibers (labelled-line model), or whether individual taste cells are sensitive to several taste modalities (across fiber pattern model). Currently, taste cells are categorized into three groups according to morphological, biochemical and physiological properties (for a review, see[1,2]). Type I cells make up about 50% of the total number of cells in a bud and are believed to have a support role, similar to glial cells in the nervous system. Type I cells wrap around other cells in the bud in a glial-like fashion [3]and express enzymes for inactivation and uptake of transmitters [4,5]. Notably, these cells have voltage-dependent outward currents, but they lack a voltage-gated inward current [6,7]. Type II cells (about 35% of the cells) possess the G protein-coupled receptors and machinery for the transduction of sweet, bitter and umami compounds. This machinery includes PLCβ2 and TRPM5; antibodies to these two proteins were previously shown to label all Type II taste cells in circumvallate taste buds [8,9]. Type II cells have voltage-gated Na^+ ^and K^+ ^channels but no voltage-gated Ca^2+ ^channels [6]. Moreover, these cells lack classical chemical synaptic contacts with gustatory nerve fibres but release ATP to communicate with adjacent cells and/or nerve endings [10-12]. Finally, type III cells have voltage-gated Na^+^, K^+ ^and Ca^2+ ^channels [6]and form conventional synapses with afferent gustatory nerve fibres [8,9]. Antibodies to SNAP-25, a presynaptic snare protein, can be used as a selective marker for Type III taste cells [13]. The role of Type III cells in the taste bud is not yet clear. They are known to release serotonin in response to stimulation of Type II cells, suggesting a role in sensory integration of the taste bud, [11,14]. However, they also respond to sour stimuli, suggesting a direct role in taste transduction [15,16].

Although it seems clear that the type II cells are responsible for the detection and transduction of sweet, bitter and umami stimuli, and the Type III cells for sour stimuli, the type of cell responding to salty stimuli is completely unknown. Salt taste transduction involves amiloride-insensitive and amiloride-sensitive pathways. The amiloride-insensitive pathway was originally proposed to be mediated by a paracellular shunt pathway, involving diffusion of Na^+ ^through tight junctions, where it interacts with basolateral channels to depolarize the cells [17,18]. More recently, TRPV1, an apical vanilloid receptor-1 variant cationic channel, was proposed as a salt receptor [19]. However, TRPV1 knockout mice retain salt sensitivity, suggesting other mechanism must contribute to salt taste.

On the other hand, it is well known that the amiloride-sensitive mechanism involves direct depolarization of taste cells by Na^+ ^permeation of epithelial sodium channels (ENaCs). This channel is expressed on the apical membrane of many epithelial cells, where it is involved in the transport of Na^+ ^across the tissue. The channel is highly selective for Na^+ ^over K^+^, is highly sensitive to amiloride (Ki = 0.1 μM), and is normally constitutively open (for review, [20]). Three homologous subunits (α, β and γ) make up the channel [20], all of which are required for normal function. All three subunits of ENaC have been identified in taste cells of rat [21-23] and mouse [24]. However, the expression of the three subunits varies in the different papillae, with more expression in fungiform than in foliate and circumvallate papillae [23,25]. The ENaCs seem to play a crucial role in the taste transduction of Na^+ ^salt since behavioural studies in rat [26,27] and in mouse [28,29] showed that amiloride decreases the taste perception of NaCl. Similarly, chorda tympani nerve recordings showed that amiloride significantly inhibits responses to NaCl in rat [30-35], hamster [36,37] and mouse [38]. Amiloride-block of ENaC channels decreases a resting Na^+ ^current in taste cells, including frog [39], rat[23,40-42], hamster [43] and mouse [44,45].

The present study investigates the functional expression of amiloride-sensitive channels in mouse fungiform taste buds. Using transgenic mice expressing GFP from the TRPM5 promoter to identify specific cell types, we report here that functional expression of amiloride-sensitive Na^+ ^channels appears to be limited to Type I taste cells, previously thought to have only a support function in mouse taste buds. Further, we have found that fungiform taste cells have a significantly smaller proportion of Type III cells and TRPM5-positive Type II cells than circumvallate taste buds, suggesting fundamental differences between fungiform and circumvallate taste buds.

## Results

### Cell type characterization

**Figure 1:**
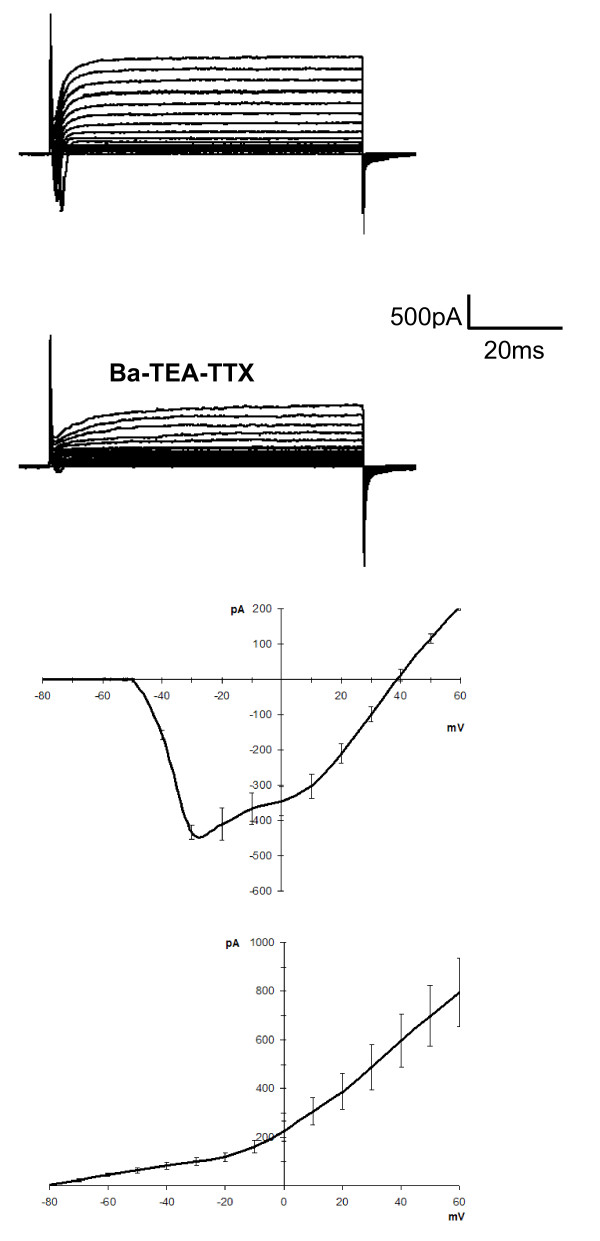
**Whole-cell voltage-gated currents in isolated TRPM5-GFP-positive fungiform taste cells**. In GFP-labeled cells expressing TRPM5 channels, depolarizing steps from -80 mV elicit voltage-gated inward and outward currents. The outward current is mostly blocked by TEA indicating the involvement of voltage-gated K^+ ^channels while the inward current was blocked by TTX indicating the involvement of voltage-gated Na^+ ^channels. Replacement of Ca^2+ ^with Ba^2+ ^did not reveal an inward current suggesting that these cells do not express voltage-gated Ca^2+ ^channels. The I-V plot is represented for both inward and outward currents in Tyrodes (n = 6 cells; mean ± sem).

**Figure 2:**
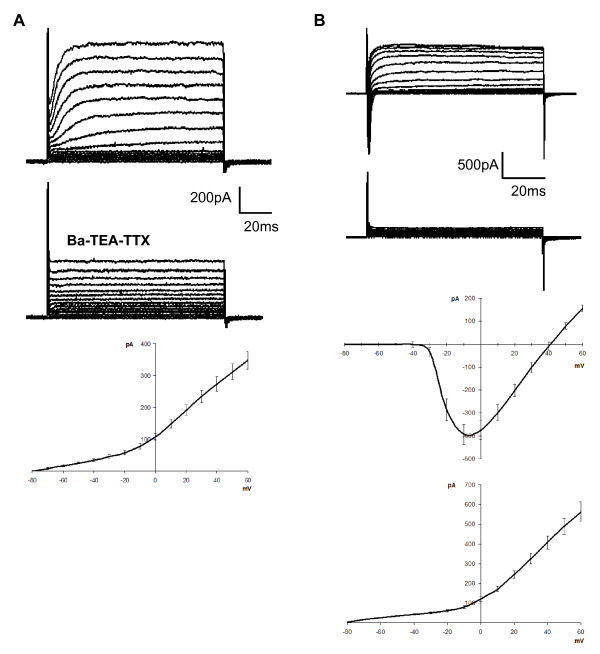
**Whole-cell voltage-gated currents in unlabeled fungiform taste cells of the TRPM5-GFP mice**. A: Some unlabeled cells (53%) exhibit only an outward current in response to depolarizing voltage steps. The Barium-TEA-TTX solution decreased the outward current indicating the involvement of voltage-dependent K^+ ^channels. The I-V plot is represented (n = 52 cells; mean ± sem). B: Other cells (46%) exhibit outward K^+ ^and inward Na^+ ^currents, but lack a voltage-dependent Ca^2+ ^(Ba^2+^) current. The I-V plot is represented for inward and outward currents (n = 45 cells; mean ± sem).

**Figure 3:**
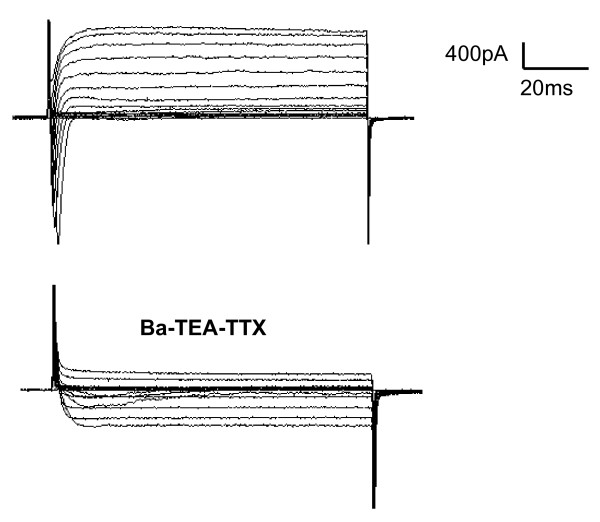
**Whole-cell voltage-gated currents in an unlabeled fungiform taste cell expressing a Ca^2+ ^current**. In one cell, showing a large inward Na^+ ^and outward current K^+ ^in response to depolarizing voltage steps, the application in of the Barium-TEA-TTX solution elicited a Ca^2+ ^current, typical of type III cells.

To identify cell types, we used TRPM5-GFP mice, which express GFP only in Type II taste cells. Based on the results of previous studies [6,7,46], we assumed that the GFP-labeled cells would be Type II cells, while the remaining unlabeled cells would be either Type I cells, which should lack voltage-dependent inward Na^+ ^current, or Type III cells, which should express voltage-gated Ca^2+ ^currents as well as Na^+ ^and K^+ ^currents. In all cases, isolated fungiform taste cells were used for recording. The presence of voltage-gated currents was examined using depolarizing steps while the cell potential was fixed at -80 mV. To identify Type III cells, we used a Barium-TEA-TTX solution, to block outward current and enhance current through voltage-gated Ca^2+ ^channels, which are present only in Type III taste cells [6]. All GFP-labeled taste cells (n = 6) had inward Na^+ ^and outward K^+ ^currents, but lacked voltage-gated Ca^2+^(Ba^2+^) currents (Figure 1), as expected from previous studies of circumvallate taste cells [6,9]. Out of a total of 98 unlabeled cells, 52 cells exhibited only voltage-gated outward currents (Figure 2A), characteristic of Type I taste cells [6]. The remaining 46 cells exhibited both inward and outward voltage gated currents (Figure 2B), but only one cell showed a Ba^2+ ^current that characterizes Type III taste cells (Figure 3). GFP-labeled cells and unlabeled cells with voltage-gated Na^+ ^and K^+ ^currents had nearly identical current profiles, with a similar voltage-dependence of activation and similar kinetics (compare Figs. 1 and 2B). Thus, we hypothesize that the unlabeled taste cells with large voltage-gated Na^+ ^and K^+ ^currents are Type II cells that lack TRPM5 expression.

### Amiloride effect on taste receptor cells

**Figure 4:**
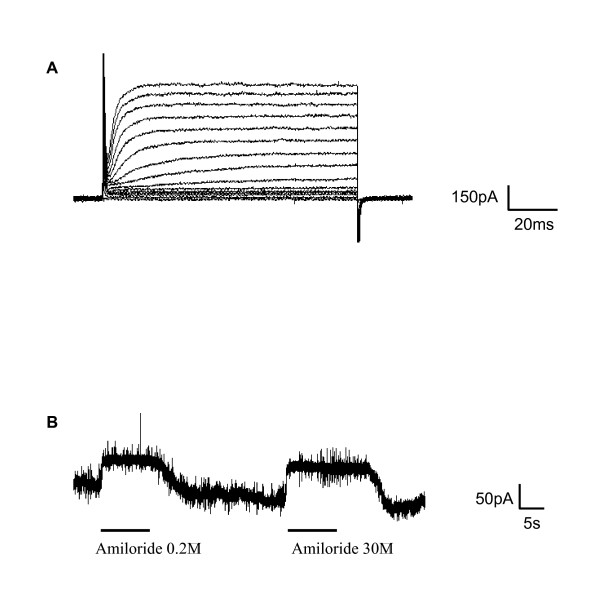
**Amiloride effect on the steady-state current in a fungiform taste cell**. A: Cells exhibiting an amiloride effect respond to depolarizing voltage steps with only outward currents. B: The application of amiloride in the bath, at 30 or 0.2 μM, decreased the steady-state current (holding potential -100 mV).

Cells were stimulated sequentially, with intervening washes, with 30 μM and 0.2 μM amiloride applied in the bath. Since taste cells were isolated, both apical and basolateral membranes were bathed with the amiloride solution. Among all recorded cells (n = 104), only 8 cells exhibited a response to amiloride. In amiloride-sensitive taste cells, amiloride decreased a steady inward current. The response varied in different cells between 25 and 160 pA (mean ± sd = 80.6 ± 51.8 with 30 μM and 43.1 ± 37.3 with 0.2 μM). Since both concentrations of amiloride were effective on the responding taste cells, these responses were likely mediated by apical amiloride-sensitive Na^+ ^channels. The small but significant (χ-square-test; χ^2 ^= 8.6; p < 0.01; df = 1) number of cells having an amiloride effect was exclusively observed in the subset of cells with only voltage-gated outward currents (Figure 4). No response to amiloride was observed in cells with voltage-gated inward currents. The addition of amiloride to either the enzymatic dissociation solution or the Tyrode's bathing taste cells during dissociation did not increase the incidence of amiloride-sensitive taste cells, suggesting that the amiloride-sensitive channels were not degraded during the taste cell isolation procedure.

### Immunocytochemistry

**Figure 5:**
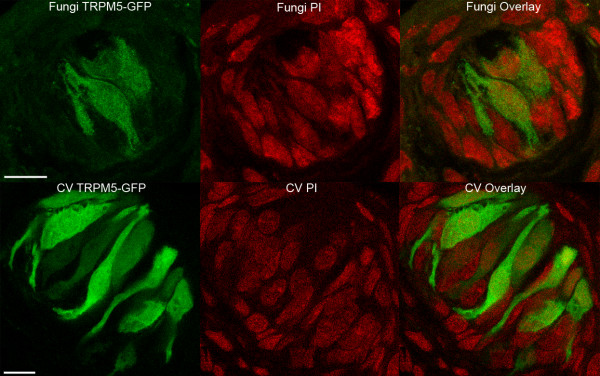
**Expression of TRPM5-GFP and Propidium Iodide in confocal images of fungiform and circumvallate taste buds**. Propidium Iodide was used to stain the nucleus of all types of cells (red) in a taste bud. Using the same section thickness, fungiform taste buds (top figures) and circumvallate taste buds (bottom figures) showed the same number of cells. Scale bar: 10 μm.

A large proportion of unlabeled taste cells exhibited large voltage-gated Na^+ ^currents, similar to TRPM5-GFP-labeled taste cells. This was unexpected, since the GFP in TRPM5-GFP mice was expected to label all Type II taste cells, based on previous studies of circumvallate taste buds [8,9]. Further, we found only one taste cell (out of 98 unlabeled taste cells) that exhibited Ca^2+ ^currents, characteristic of Type III taste cells. In circumvallate taste buds, approximately 15–20% of taste cells exhibit increases in Ca^2+ ^due to K^+ ^depolarization [15]. To determine if fungiform taste buds have a different complement of cell types compared with circumvallate taste buds, we examined fungiform and circumvallate taste buds with immunocytochemical markers characteristic of Type II and Type III taste cells (i.e., PLCβ2 for Type II cells and SNAP-25 for Type III cells). Section thickness was comparable in fungiform and circumvallate taste buds (Student's t test; NS; df = 76; Table 1). Out of 40 sections of fungiform taste buds examined, the number of GFP-labeled taste cells per section ranged from 0 to 6, with a mean of 2.8 ± 1.3 (mean ± sd). In sections of circumvallate taste buds, GFP labelled taste cells were significantly more abundant (Student t test; p < 0.01; df = 80) relative to fungiform taste buds (Table 1). Out of 42 sections examined, GFP-labeled circumvallate taste cells ranged from 3 to 15, with a mean of 7.5 ± 2.7 (mean ± sd). Further, we used propidium iodide to reveal the total number of cells per section (section thickness = 4 μm). No significance difference (Student's t test; NS; df = 22) was observed between the total number of cells in fungiform sections (mean ± sd = 13.9 ± 3.3; n = 11; Figure 5 top) and circumvallate sections (mean ± sd = 15.5 ± 5; n = 13; Figure 5 bottom), indicating that the difference was not due to variations in taste bud size between circumvallate and fungiform taste buds.

**Table 1:** Comparison of fungiform and circumvallate taste buds in TRPM5-GFP mice.

	Bud diameter (μm)	Section thickness (μm)	Number of GFP cells	Number of PLCβ2 cells	Number of SNAP25 cells
Fungiform taste buds	40.1 ± 4.2 (n = 18)	12.8 ± 5.6 (n = 40)	2.8 ± 1.3 (n = 40)	4.2 ± 1.9 (n = 20)	1.6 ± 1.1 (n = 20)
Circumvallate taste buds	43.3 ± 7.4 (n = 29)	14.2 ± 6.0 (n = 38)	7.5 ± 2.7 (n = 42)	6.0 ± 1.9 (n = 20)	4.4 ± 1.8 (n = 22)

The diameter of the taste buds was similar in fungiform and circumvallate taste buds, indicating similar numbers of cells in each type of taste bud. However, the number of GFP-positive cells, SNAP-25-ir and PLCβ2-ir cells was significantly higher in the circumvallate taste buds than in the fungiform taste buds (Student's t test; p < 0.01; df = 80 for GFP, df = 40 for SNAP-25 and df = 38 for PLCβ2). Note that in the fungiform taste buds, the PLCβ2-ir cells are significantly more numerous than the TRPM5-GFP cells (Student's t test; p < 0.01; df = 19).

#### PLCβ2 and GFP

**Figure 6:**
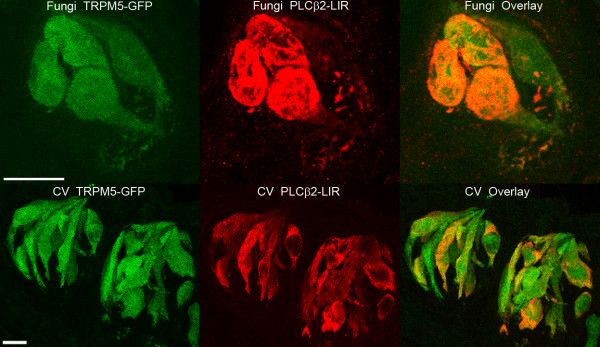
**Expression of TRPM5-GFP and PLCβ2 in confocal images of fungiform and circumvallate taste buds**. The top figures illustrate the expression of GFP under the control of the TRPM5 promoter (green) and PLCβ2-ir (red) in fungiform taste buds. The bottom figures illustrate labeling in circumvallate taste buds. Note that in circumvallate taste buds there are far more TRPM5-GFP cells relative to fungiform taste buds, however PLCβ2 and TRPM5-GFP were generally co-localized in both fungiform and circumvallate taste buds. Each figure represents merged images from a Z-series. Scale bar: 10 μm.

Sections of fungiform papillae from TRPM5-GFP mice were processed for PLCβ2 immunoreactivity (PLCβ2-ir) and observed with laser scanning confocal microscopy. The majority of taste cells expressing GFP also expressed PLCβ2-ir (Figure 6; Table 1), although a few taste cells expressing PLCβ2-ir lacked GFP expression. In circumvallate taste buds, all PLCβ2 labelled taste cells co-expressed TRPM5-GFP (n = 121; Figure 6), consistent with previous studies [9]. However, we did observe a few TRPM5-GFP cells that lacked PLCβ2 expression. Nonetheless, these data extend and confirm previous studies suggesting that TRPM5-GFP expression is a reliable reporter of taste cells that express the PLC signaling pathway in both fungiform and circumvallate taste buds [9]. These data, taken together with the electrophysiological data, suggest that a significant proportion of fungiform taste cells with electrophysiological properties of Type II taste cells lack PLC signaling components.

#### SNAP-25 and GFP

**Figure 7:**
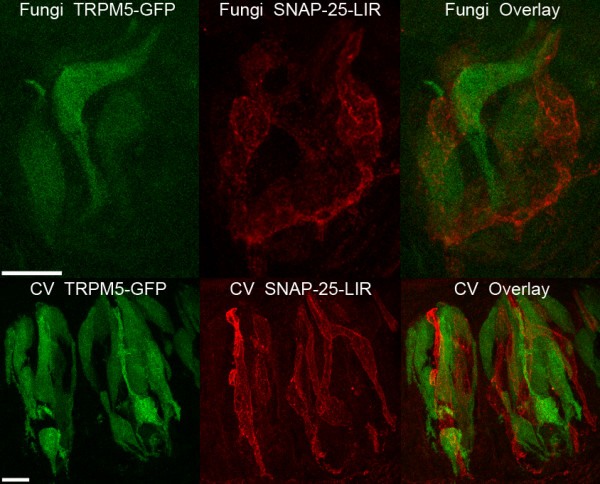
**Expression of TRPM5-GFP and SNAP-25 in confocal images of fungiform and circumvallate taste buds**. The top figures illustrate the expression of GFP under the control of the TRPM5 promoter (green) and SNAP-25-ir (red) in fungiform taste buds and the bottom figures illustrate labeling in circumvallate taste buds. No co-localization was observed between TRPM5-GFP and SNAP-25-ir. The number of SNAP-25-ir and GFP-labeled cells was larger in circumvallate than in fungiform taste buds. Each figure represents merged images from a Z-series. Scale bar: 10 μm.

Evaluation of 98 randomly-selected unlabeled taste cells revealed that only a single taste cell expressed voltage-gated Ca^2+ ^currents, characteristic of Type III taste cells. To determine if this was due to poor survivability of isolated Type III cells, or to a genuine difference in the distribution of Type III cells in fungiform taste buds, we labelled sections of fungiform taste buds with an antibody against SNAP-25, a presynaptic protein specifically expressed in Type III taste cells [13]. Out of 20 sections examined, SNAP-25-ir-labeled cells ranged from 0 to 4, with a mean of 1.6 ± 1.1 (mean ± sd) (Figure 7; Table 1). In comparison, sections of circumvallate taste buds averaged 4.4 ± 1.8 (mean ± sd) immunoreactive cells, similar to what was observed previously [9]. These differences between papillae are significantly different (Student's t test; p < 0.01; df = 40), indicating that fungiform taste cells have fewer Type III taste cells than circumvallate papillae. As expected from previous studies [9,47], SNAP-25-ir expression was completely independent of TRPM5-GFP expression, suggesting that taste cells expressing PLC signaling components do not make conventional synapses with the nervous system in either fungiform or circumvallate taste buds.

## Discussion

In the present study, physiological responses to amiloride were examined in defined subsets of mouse fungiform taste cells. The principal finding is that amiloride-sensitive Na^+ ^channels, thought to be required for amiloride-sensitive NaCl transduction, appear to be functionally expressed in taste cells lacking voltage-dependent inward currents. These cells are likely to be the Type I taste cells, thought previously to have only a support function in the taste bud [3-5]. However, it is also possible that cells lacking inward currents are developing taste cells that have not yet reached the taste pore. Developing taste cells have slowly activating outward currents compared to mature receptor cells [48]. Indeed, we recorded from a small number of taste cells that had slowly activating outward currents typical of developing taste cells, but none of these exhibited an amiloride effect. Interestingly, only 7.7% of the total cells recorded from were amiloride-sensitive. The proportion of cells responding to amiloride seems low compared to previous studies showing that about 65% of the cells responded to amiloride in mouse taste cells maintained in an intact taste bud [44]. However, it is now well-know that taste cells communicate between each other [49] and that the information contained in one cell can be transferred to adjacent cell(s). Hence several cells can respond to NaCl even if they do not possess the ENaC. Nevertheless, using in situ hybridization, Shigemura et al. (2005) showed that, in mouse, only 2 to 4 cells express the different ENaC subunits in a fungiform taste bud. This observation correlates with our findings and suggests that only a small proportion of taste cells express amiloride-sensitive channels in mouse. Further, rat and mouse show numerous morphological differences [50] as well as physiological differences. Indeed, responses to amiloride occurred in rat taste cells with both inward and outward voltage-gated currents (Type II cells) but not in cells showing a Ca^2+ ^current (Type III cells) [42]. However, these authors did not record from cells with only outward voltage-gated currents (likely Type I cells). It would be predictable that many rat type I cells would be sensitive to amiloride since Doolin and Gilbertson showed that 75% of rat fungiform taste cells expressing only outward currents are sensitive to amiloride. Thus, our results disclose a remarkable difference between species and could explain the different detection threshold measured in rat and in mouse, i.e: between 0.001 M and 0.002 M in Wistar rat [51] compared to 0.065 M in C57BL/6J mouse [28].

Because the cells were isolated in this study, and amiloride was bath-applied, it was not possible to tell whether the amiloride-sensitive Na^+ ^channels were localized to the apical membrane. However, amiloride-sensitive cells responded to the lowest concentration of amiloride (0.2 μM), which corresponds to the defined Ki for apical ENaCs [40]. Basolateral amiloride-sensitive Na^+ ^channels are less sensitive to amiloride (Ki = 0.56 μM) [52]. One could propose that the enzymatic treatment used to isolate the taste bud cells as well as the high concentration of Na^+ ^ions in the bath solution could degrade or desensitize the amiloride-sensitive channels [40]. However, the use of amiloride in the enzymatic treatment solution or the bathing medium did not enhance the occurrence of amiloride responses, suggesting that the channels are functional. Moreover, it seems unlikely that the enzymatic treatment degrades the channels in a particular type of cell and not in another type of cell.

The observation of responses to amiloride in cells with only voltage-gated K^+ ^channels raises the question of how amiloride-sensitive taste information is transmitted to the nervous system. According to morphological studies, type I cells do not possess synaptic contacts [1,53] or subsurface-cisternae, which have been proposed to be involved in activation of afferent nerve fibers [8]. Besides, the absence of voltage-gated Na^+ ^channels in the amiloride-sensitive cells would appear to eliminate the cell's ability to produce action potentials, however, the cells should depolarize in response to NaCl. Recently, Huang *et al.*[14] showed that Pannexin 1 (Px1) hemichannel mRNA was detected in about half of the cells expressing NTPDaseII, a marker for glial-like cells (Type I cells). Since the presence of Px1 channels is believed to underlie ATP release in the taste bud, which is required for salt taste transduction [10], Type I cells may release ATP to signal to the nervous system. Further studies will be needed to determine if Px1 channels are required for transmitting salt taste information to the nervous system, either directly, or via other cells in the taste bud.

Another interesting finding in this study is that fungiform taste buds appear to have a different complement of taste cell types than circumvallate papillae. TRPM5-GFP mice were used to identify the cells expressing the TRPM5 channel, presumably found in all type II cells, however, GFP fluorescence was observed only in a few cells in each taste bud of fungiform papillae, while it was observed in many cells of circumvallate taste buds. Further, many of the unlabeled cells exhibited physiological criteria used to characterize cells as a Type II cell (i.e): presence of voltage-gated Na^+ ^and K^+ ^channels and lack of voltage-gated Ca^2+ ^channels [6,9,47]. Unlike circumvallate taste cells, PLCβ2 was expressed in some non-TRPM5-GFP fungiform cells, although the number of these taste cells is not sufficient to account for the large percentage of unlabeled taste cells expressing voltage-dependent Na^+ ^currents. These observations suggest that, in mouse fungiform taste buds, only a subset of cells with Type II cell membrane properties expresses PLC signalling components. The function of the PLCβ2-independent cells with voltage-gated Na^+ ^channels is unclear. Many cells in intact fungiform taste buds are electrically excitable, and generate action potentials to apically-applied stimuli representing all the taste qualities, including salt [54]. It is possible that these PLCβ2-independent cells integrate signals transduced by other cells in the taste bud, including Type I cells.

Fungiform taste cells also have a different complement of Type III taste cells than circumvallate taste buds. The proportion of SNAP-25 labeled cells is higher in circumvallate taste buds than in fungiform taste buds. This correlates with the small number of synapses observed ultrastructurally in mouse fungiform taste buds compared to foliate and vallate taste buds [55]. These observations also correspond with our inability to randomly find taste cells expressing voltage-gated Ca^2+ ^currents in the unlabeled taste cells of the TRPM5-GFP mice.

This study, taken together with previous studies, suggests that at least in fungiform taste buds, separate subsets of taste cells are specialized for transducing different taste qualities. Subsets of Type II cells with PLC signaling components mediate the transduction of sweet, umami, or bitter compounds, while a different subset, likely of Type III cells, mediates sour transduction [56]. We now show that a subset of Type I cells expresses functional ENaC channels, involved in the transduction of amiloride-sensitive, Na^+ ^specific salt taste. In addition, we show that a large subset of taste cells with Type II cell membrane properties lacks expression of PLC signaling components. Whether these cells are involved in the transduction of tastants, or in signal processing in the taste bud awaits further investigation.

## Conclusion

The principal finding in this study is that amiloride-sensitive Na^+ ^channels, required for Na^+ ^salt transduction, are located in fungiform taste cells that lack voltage-dependent inward currents. These taste cells, the so-called Type I taste cells, were previously thought to provide only a support function in the taste bud. These results raise questions about how Na^+ ^salt taste information in transmitted to the nervous system. We also provide evidence that fungiform taste buds have a different complement of cell types than circumvallate taste buds, based on electrophysiological and immunocytochemical criteria. Many electrically excitable "Type II" cells in fungiform taste buds lack PLC signaling components, which are present in all Type II cells of circumvallate taste buds. Further, fungiform taste buds have significantly fewer Type III cells than circumvallate taste buds.

## Methods

### Patch clamp recordings

#### Taste bud isolation

Adult male and female transgenic mice expressing GFP from the TRPM5 promoter (TRPM5-GFP; [9]) were used in all experiments. These mice have a C57BL/6J background. Animals were cared for in compliance with the Colorado State University Animal Care and Use Committee. Animals were sacrificed with CO_2 _and cervical dislocation. The anterior part of the tongue containing the fungiform papillae was removed and the taste cells were isolated as previously described by Behe *et al.*[57]. Briefly, 0.1 ml of a mixture of enzymes containing Dispase II (3 mg/ml; Roche, Indianapolis, IN), collagenase B (0.7 mg/ml; Roche, Indianapolis, IN), trypsin inhibitor (1 mg/ml; Sigma, Saint Louis, MO) and elastase (0.05 mg/ml, Worthington, Lakewood, NJ) diluted in a Tyrode's solution was injected under the lingual epithelium. As previously proposed [40], this enzymatic treatment could degrade the amiloride-sensitive channels. In a few experiments, 1 μM amiloride was hence added in the enzymatic solution to prevent the degradation, or 30 μM was added to the Ca-free Tyrode's during the dissociation procedure. After 30 minutes incubation in Ca-free Tyrode's, the epithelium was peeled and incubated for 5 minutes in Ca-free Tyrode's. Gentle suction with a glass capillary pipette removed fungiform taste cells that were subsequently pipetted onto Poly-L-Lysine-coated slides (Sigma, Saint Louis, US).

#### Patch clamp recordings

The whole-cell configuration of the patch-clamp technique [58] was used in this study to characterize the voltage-gated currents in taste cells and to detect a potential amiloride effect. Electrodes were pulled from borosilicate capillaries glass (LE16, Dagan Corporation, Minneapolis, MN) using a horizontal micropipette puller (P97, Sutter Instrument, Novato, CA). Pipette resistances were 10–12 MΩ when filled with KGluconate intracellular solution. Recordings were performed using an Axopatch 1D amplifier and Pclamp 9 software (Axon instruments, Foster City, CA). Signals were filtered at 5 kHz. In the whole-cell mode, membrane capacitance was partially compensated. Taste cells were depolarized in 10 mV steps from -60 to +60 mV from a holding potential of -80 mV; each step was 100 ms in duration. The steady-state current was also recorded while cells were maintained at -100 mV. Cells showing a large leak current (>150 pA) at -80 mV were not considered since this leak current could mask an amiloride effect. Leak currents were subtracted off-line prior to constructing I/V plots.

#### Solutions

Normal Tyrode's contained (in mM): 140 NaCl; 5 KCl; 4 CaCl_2_; 1 MgCl_2_; 10 HEPES; 10 glucose; 1 Na Pyruvate; pH adjusted to 7.4 with NaOH. Ca-free Tyrode's solution contained (in mM): 140 NaCl; 5 KCl; 10 HEPES; 10 glucose; 1 Na Pyruvate; 2 EGTA; 2 BAPTA; pH adjusted to 7.4 with NaOH. To reveal the presence of voltage-gated Ca^2+ ^currents, the bath solution contained (in mM): 10 BaCl_2_; 136 TEA; 2.10^-4 ^TTX; 1 MgCl_2_; 10 HEPES; 10 glucose and 1 Na pyruvate; pH was adjusted to 7.4 with NaOH. The presence of functional amiloride-sensitive Na^+ ^channels was assessed by bath application of amiloride (30 μM and 0.2 μM) diluted in Tyrode's. All solution were delivered in the bath by gravity pressure at a 10 ml/min flow rate using a perfusion system (Warner Instruments, Hamden, CT). Recording pipettes were filled with an intrapipette solution containing the following (in mM): 130 Kgluconate; 10 KCl; 2 MgCl_2_; 1 CaCl_2_; 10 HEPES; 11 EGTA; 1 ATP; 0,4 GTP; pH adjusted to 7,4 with KOH. Chemicals were purchased from Sigma Corporation (St. Louis, MO).

### Immunocytochemistry

#### Tissue preparation

Mice were killed with CO_2 _and cervical dislocation. Tongues were removed and placed in 4% paraformaldehyde (Electron Microscopy Services, Ft Washington, PA) in 0.1 M phosphate buffer (pH 7.2) for 1–3 hours. For cryoprotection, tongues were put in 20% sucrose-phosphate buffer and placed at 4°C overnight. Forty micrometer sections were cut from fungiform and circumvallate papillae on a cryostat (Leitz 1729) and collected in 0.1 M phosphate buffered saline (PBS, pH 7.2). Sections were washed three times in PBS for 10 minutes each at room temperature and incubated for 2 hours in blocking solution (0.3% Triton X-100, 1% normal goat serum, 1% bovine serum albumin in PBS).

#### Immunocytochemistry

After blocking, sections were incubated with either anti-SNAP-25 (1:200) (Rabbit, Calbiochem, SanDiego, CA) or anti-PLCβ2 (1:1000) (Rabbit, Santa Cruz Biotechnology, Santa Cruz, CA) in blocking solution and placed overnight at 4°C. For each experiment, some sections were processed without the primary antibody to control for non-specific labelling of the secondary antibody. The omission of the primary antibodies resulted in no immunoreactivity for either primary antibody. Sections were then washed three times in PBS for 10 minutes each at room temperature and incubated for 2 hours in Cy-5 anti-rabbit (1:400) (Jackson ImmunoResearch, West Grove, PA). Sections were then washed three times in PBS for 10 minutes each at room temperature and mounted on slides using Flouromount-G (Southern Biotechnology, Birmingham, AL). Some sections were labeled with propidium iodide (a nuclear marker) and treated as follows: after blocking, sections were rinsed three times in PBS for 10 minutes each and then incubated in 0.1 M PBS containing 10 mg/ml MgCl_2 _and 250 μg/ml RNaseA (Sigma, St Louis, MO) for 30 minutes at 35°C. After three rinses in PBS, sections were incubated in 0.5 μg/ml propidium iodide (Sigma, St Louis, MO) for 1 min, rinsed three more times and finally mounted on slides. Images were acquired using an Olympus FVX-IHRT Fluoview Confocal Laser Scanning Microscope (Tokyo, Japan) and the Fluoview software. Images were processed and printed using Adobe Photoshop CS2 software.

## Authors' contributions

Immunochemistry was done by AV and TRC. AV and SCK performed the patch clamp recordings and wrote the manuscript.

## References

[B1] Finger TE (2000). Cell Biology of Taste Epithelium. In Neurobiology of Taste and Smell, 2nd edition Edited by Finger, TE, Silver, WL, Restrepo, D New York: Wiley Liss.

[B2] Kinnamon SC (2007). Taste Transduction. In Handbook of Senses Volume 1 Edited by Firestein, S, Smith, D, Shepherd, G In Press.

[B3] Pumplin DW, Yu C, Smith DV (1997). Light and dark cells of rat vallate taste buds are morphologically distinct cell types. J Comp Neurol.

[B4] Lawton DM, Furness DN, Lindemann B, Hackney CM (2000). Localization of the glutamate-aspartate transporter, GLAST, in rat taste buds. Eur J Neurosci.

[B5] Bartel DL, Sullivan SL, Lavoie EG, Sevigny J, Finger TE (2006). Nucleoside triphosphate diphosphohydrolase-2 is the ecto-ATPase of type I cells in taste buds. J Comp Neurol.

[B6] Medler KF, Margolskee RF, Kinnamon SC (2003). Electrophysiological characterization of voltage-gated currents in defined taste cell types of mice. J Neurosci.

[B7] Romanov RA, Kolesnikov SS (2006). Electrophysiologically identified subpopulations of taste bud cells. Neurosci Lett.

[B8] Clapp TR, Yang R, Stoick CL, Kinnamon SC, Kinnamon JC (2004). Morphologic characterization of rat taste receptor cells that express components of the phospholipase C signaling pathway. J Comp Neurol.

[B9] Clapp TR, Medler KF, Damak S, Margolskee RF, Kinnamon SC (2006). Mouse taste cells with G protein-coupled taste receptors lack voltage-gated calcium channels and SNAP-25. BMC Biol.

[B10] Finger TE, Danilova V, Barrows J, Bartel DL, Vigers AJ, Stone L, Hellekant G, Kinnamon SC (2005). ATP signaling is crucial for communication from taste buds to gustatory nerves. Science.

[B11] Huang YJ, Maruyama Y, Dvoryanchikov G, Pereira E, Chaudhari N, Roper SD (2007). The role of pannexin 1 hemichannels in ATP release and cell-cell communication in mouse taste buds. Proc Natl Acad Sci U S A.

[B12] Romanov RA, Rogachevskaja OA, Bystrova MF, Jiang P, Margolskee RF, Kolesnikov SS (2007). Afferent neurotransmission mediated by hemichannels in mammalian taste cells. Embo J.

[B13] Yang R, Crowley HH, Rock ME, Kinnamon JC (2000). Taste cells with synapses in rat circumvallate papillae display SNAP-25-like immunoreactivity. J Comp Neurol.

[B14] Huang YJ, Maruyama Y, Lu KS, Pereira E, Plonsky I, Baur JE, Wu D, Roper SD (2005). Mouse taste buds use serotonin as a neurotransmitter. J Neurosci.

[B15] Richter TA, Caicedo A, Roper SD (2003). Sour taste stimuli evoke Ca2+ and pH responses in mouse taste cells. J Physiol.

[B16] Tomchik SM, Berg S, Kim JW, Chaudhari N, Roper SD (2007). Breadth of tuning and taste coding in mammalian taste buds. J Neurosci.

[B17] Elliott EJ, Simon SA (1990). The anion in salt taste: a possible role for paracellular pathways. Brain Res.

[B18] Ye Q, Heck GL, DeSimone JA (1991). The anion paradox in sodium taste reception: resolution by voltage-clamp studies. Science.

[B19] Lyall V, Heck GL, Vinnikova AK, Ghosh S, Phan TH, Alam RI, Russell OF, Malik SA, Bigbee JW, DeSimone JA (2004). The mammalian amiloride-insensitive non-specific salt taste receptor is a vanilloid receptor-1 variant. J Physiol.

[B20] Garty H, Palmer LG (1997). Epithelial sodium channels: function, structure, and regulation. Physiol Rev.

[B21] Simon SA, Holland VF, Benos DJ, Zampighi GA (1993). Transcellular and paracellular pathways in lingual epithelia and their influence in taste transduction. Microsc Res Tech.

[B22] Li XJ, Blackshaw S, Snyder SH (1994). Expression and localization of amiloride-sensitive sodium channel indicate a role for non-taste cells in taste perception. Proc Natl Acad Sci U S A.

[B23] Lin W, Finger TE, Rossier BC, Kinnamon SC (1999). Epithelial Na+ channel subunits in rat taste cells: localization and regulation by aldosterone. J Comp Neurol.

[B24] Shigemura N, Islam AA, Sadamitsu C, Yoshida R, Yasumatsu K, Ninomiya Y (2005). Expression of amiloride-sensitive epithelial sodium channels in mouse taste cells after chorda tympani nerve crush. Chem Senses.

[B25] Kretz O, Barbry P, Bock R, Lindemann B (1999). Differential expression of RNA and protein of the three pore-forming subunits of the amiloride-sensitive epithelial sodium channel in taste buds of the rat. J Histochem Cytochem.

[B26] Roitman MF, Bernstein IL (1999). Amiloride-sensitive sodium signals and salt appetite: multiple gustatory pathways. Am J Physiol.

[B27] Geran LC, Spector AC (2000). Amiloride increases sodium chloride taste detection threshold in rats. Behav Neurosci.

[B28] Eylam S, Spector AC (2002). The effect of amiloride on operantly conditioned performance in an NaCl taste detection task and NaCl preference in C57BL/6J mice. Behav Neurosci.

[B29] Eylam S, Spector AC (2003). Oral amiloride treatment decreases taste sensitivity to sodium salts in C57BL/6J and DBA/2J mice. Chem Senses.

[B30] Heck GL, Mierson S, DeSimone JA (1984). Salt taste transduction occurs through an amiloride-sensitive sodium transport pathway. Science.

[B31] Hill DL, Bour TC (1985). Addition of functional amiloride-sensitive components to the receptor membrane: a possible mechanism for altered taste responses during development. Brain Res.

[B32] DeSimone JA, Ferrell F (1985). Analysis of amiloride inhibition of chorda tympani taste response of rat to NaCl. Am J Physiol.

[B33] Ninomiya Y, Funakoshi M (1988). Amiloride inhibition of responses of rat single chorda tympani fibers to chemical and electrical tongue stimulations. Brain Res.

[B34] Heck GL, Persaud KC, DeSimone JA (1989). Direct measurement of translingual epithelial NaCl and KCl currents during the chorda tympani taste response. Biophys J.

[B35] Lundy RF, Contreras RJ (1997). Temperature and amiloride alter taste nerve responses to Na+, K+, and NH+4 salts in rats. Brain Res.

[B36] Herness MS (1987). Effect of amiloride on bulk flow and iontophoretic taste stimuli in the hamster. J Comp Physiol [A].

[B37] Hettinger TP, Frank ME (1990). Specificity of amiloride inhibition of hamster taste responses. Brain Res.

[B38] Ninomiya Y, Sako N, Funakoshi M (1989). Strain differences in amiloride inhibition of NaCl responses in mice, Mus musculus. J Comp Physiol [A].

[B39] Avenet P, Lindemann B (1988). Amiloride-blockable sodium currents in isolated taste receptor cells. J Membr Biol.

[B40] Doolin RE, Gilbertson TA (1996). Distribution and characterization of functional amiloride-sensitive sodium channels in rat tongue. J Gen Physiol.

[B41] Kossel AH, McPheeters M, Lin W, Kinnamon SC (1997). Development of membrane properties in taste cells of fungiform papillae: functional evidence for early presence of amiloride-sensitive sodium channels. J Neurosci.

[B42] Bigiani A, Cuoghi V (2007). Localization of amiloride-sensitive sodium current and voltage-gated calcium currents in rat fungiform taste cells. J Neurophysiol.

[B43] Gilbertson TA, Fontenot DT (1998). Distribution of amiloride-sensitive sodium channels in the oral cavity of the hamster. Chem Senses.

[B44] Miyamoto T, Fujiyama R, Okada Y, Sato T (1999). Strain difference in amiloride-sensitivity of salt-induced responses in mouse non-dissociated taste cells. Neurosci Lett.

[B45] Miyamoto T, Miyazaki T, Fujiyama R, Okada Y, Sato T (2001). Differential transduction mechanisms underlying NaCl- and KCl-induced responses in mouse taste cells. Chem Senses.

[B46] Bigiani A, Cristiani R, Fieni F, Ghiaroni V, Bagnoli P, Pietra P (2002). Postnatal development of membrane excitability in taste cells of the mouse vallate papilla. J Neurosci.

[B47] DeFazio RA, Dvoryanchikov G, Maruyama Y, Kim JW, Pereira E, Roper SD, Chaudhari N (2006). Separate populations of receptor cells and presynaptic cells in mouse taste buds. J Neurosci.

[B48] Mackay-Sim A, Delay RJ, Roper SD, Kinnamon SC (1996). Development of voltage-dependent currents in taste receptor cells. J Comp Neurol.

[B49] Roper SD (2006). Cell communication in taste buds. Cell Mol Life Sci.

[B50] Ma H, Yang R, Thomas SM, Kinnamon JC (2007). Qualitative and quantitative differences between taste buds of the rat and mouse. BMC Neurosci.

[B51] Clarke SN, Koh MT, Bernstein IL (2001). NaCl detection thresholds: comparison of Fischer 344 and Wistar rats. Chem Senses.

[B52] Mierson S, Olson MM, Tietz AE (1996). Basolateral amiloride-sensitive Na+ transport pathway in rat tongue epithelium. J Neurophysiol.

[B53] Royer SM, Kinnamon JC (1994). Application of serial sectioning and three-dimensional reconstruction to the study of taste bud ultrastructure and organization. Microsc Res Tech.

[B54] Yoshida R, Yasumatsu K, Shigemura N, Ninomiya Y (2006). Coding channels for taste perception: information transmission from taste cells to gustatory nerve fibers. Arch Histol Cytol.

[B55] Kinnamon JC, Henzler DM, Royer SM (1993). HVEM ultrastructural analysis of mouse fungiform taste buds, cell types, and associated synapses. Microsc Res Tech.

[B56] Chandrashekar J, Hoon MA, Ryba NJ, Zuker CS (2006). The receptors and cells for mammalian taste. Nature.

[B57] Behe P, DeSimone JA, Avenet P, Lindemann B (1990). Membrane currents in taste cells of the rat fungiform papilla. Evidence for two types of Ca currents and inhibition of K currents by saccharin. J Gen Physiol.

[B58] Hamill OP, Marty A, Neher E, Sakmann B, Sigworth FJ (1981). Improved patch-clamp techniques for high-resolution current recording from cells and cell-free membrane patches. Pflugers Arch.

